# Metabolomic Signatures of Brainstem in Mice following Acute and Subchronic Hydrogen Sulfide Exposure

**DOI:** 10.3390/metabo14010053

**Published:** 2024-01-14

**Authors:** Dong-Suk Kim, Cristina M. Santana Maldonado, Cecilia Giulivi, Wilson Kiiza Rumbeiha

**Affiliations:** 1 Department of Molecular Biosciences, School of Veterinary Medicine, UC Davis, Davis, CA 95616, USA; dskkim@ucdavis.edu (D.-S.K.); csantanamaldonado@mriglobal.org (C.M.S.M.); cgiulivi@ucdavis.edu (C.G.); 2 MRI Global, Kansas City, MO 64110, USA

**Keywords:** hydrogen sulfide, metabolomics, brainstem, brain, metabolism, biomarkers, neurotransmitters, branched-chain amino acids, polyunsaturated fatty acids

## Abstract

Hydrogen sulfide (H_2_S) is an environmental toxicant of significant health concern. The brain is a major target in acute H_2_S poisoning. This study was conducted to test the hypothesis that acute and subchronic ambient H_2_S exposures alter the brain metabolome. Male 7–8-week-old C57BL/6J mice were exposed by whole-body inhalation to 1000 ppm H_2_S for 45 min and euthanized at 5 min or 72 h for acute exposure. For subchronic study, mice were exposed to 5 ppm H_2_S 2 h/day, 5 days/week for 5 weeks. Control mice were exposed to room air. The brainstem was removed for metabolomic analysis. Enrichment analysis showed that the metabolomic profiles in acute and subchronic H_2_S exposures matched with those of cerebral spinal fluid from patients with seizures or Alzheimer’s disease. Acute H_2_S exposure decreased excitatory neurotransmitters, aspartate, and glutamate, while the inhibitory neurotransmitter, serotonin, was increased. Branched-chain amino acids and glucose were increased by acute H_2_S exposure. Subchronic H_2_S exposure within OSHA guidelines surprisingly decreased serotonin concentration. In subchronic H_2_S exposure, glucose was decreased, while polyunsaturated fatty acids, inosine, and hypoxanthine were increased. Collectively, these results provide important mechanistic clues for acute and subchronic ambient H_2_S poisoning and show that H_2_S alters brainstem metabolome.

## 1. Introduction

The brain is a highly complex organ that regulates many body functions. To perform these roles, it relies on complex metabolic processes which are energy dependent [1,2]. Consequently, the brain is susceptible to toxic environmental chemicals that target mitochondria and impair energy metabolism. An example of such chemicals is hydrogen sulfide (H_2_S), a potent gas produced endogenously and in the environment. Endogenously produced H_2_S plays key roles in brain signaling and is essential for normal brain physiologic functions. However, excessive amounts of H_2_S in the brain, either from aberrant endogenous metabolic pathways (genetic defects) or environmental exposures, leads to neuronal dysfunction, including neurodegeneration [3,4,5,6,7,8,9,10]. Ethylmalonic encephalopathy (OMIM 602473), a life-limiting disease, is caused by a genetic defect in H_2_S metabolism in mitochondria, resulting in chronic excessive H_2_S exposure in the brain [11]. 

Studies from our laboratory showed that acute exogenous H_2_S exposure induces transcriptomic and proteomic changes in the brainstem, thalamus, and other brain regions [3,5,6,7,8,9,12,13]. A wealth of literature shows that H_2_S impairs mitochondrial function, specifically targeting cytochrome c oxidase (Complex IV) in the electron transport chain, thus inhibiting ATP production [14,15,16,17]. Chemical hypoxia and/or energy deficits are linked to a loss of central respiratory drive, convulsions, and death following acute H_2_S exposures [18,19]. In cases without a fatal outcome, it is likely that deficits in brain metabolism may cause immediate, intermediate, delayed, and long-term neurotoxicity following acute or chronic H_2_S exposure. It is possible that some of these H_2_S-induced toxicological changes may predispose exposed individuals to other neurological conditions, including neurodegenerative conditions via gene–environment interactions. To our knowledge, despite the well-known impact of H_2_S on cellular metabolism, there are no metabolomic studies on the brainstem upon acute and chronic H_2_S exposures. This is a knowledge gap curtailing our understanding of toxic mechanisms of H_2_S-induced neurotoxicity. 

We postulated that metabolic signatures of the brainstem could be observed in an inhalation whole-mouse model because human exposure to H_2_S gas is typically via inhalation. For this project, we focused on the brainstem because this region has been previously shown to undergo neurodegeneration following acute H_2_S exposure and also because it controls vital autonomic functions such as breathing [3]. Impaired breathing is widely implicated as a cause of death following acute H_2_S poisoning [18]. It was also previously reported that H_2_S preferentially accumulates in the brainstem region [20]. Moreover, the exact underlying mechanisms by which H_2_S suppresses breathing, causing death, remain to be elucidated. Therefore, understanding the metabolomic changes in the brainstem following H_2_S exposure will advance our knowledge of the mechanisms underlying H_2_S-induced neurotoxicity, neuropathology, and death.

## 2. Materials and Methods

### 2.1. Animals

All animal studies were approved by the Institutional Animal Care and Use Committee (IACUC) of Iowa State University (ISU) and the University of California at Davis (UC Davis). IACUC-18-136, February 2016, and IACUC-21819, October 2020, were approved by ISU and UC Davis, respectively. Care and use of animals were performed in accordance with the IACUC, and animals were treated humanely and handled with care. We also followed the ARRIVE guidelines in the design and execution of this study [21].

Briefly, 7–8-week-old male C57BL/6J mice were purchased from Jackson Laboratories (Sacramento, CA and Bar Harbor, ME, USA). Only male mice were used because previous studies in our laboratory found male mice to be more sensitive to acute H_2_S poisoning than females [9]. Mice were randomly assigned to different groups and housed in Laboratory Animal Resources at the College of Veterinary Medicine, ISU, and in the Teaching and Research Animal Care Services facility (TRACS) at the School of Veterinary Medicine, UC Davis, with a 12:12 h light and dark cycle. Room temperature and relative humidity were maintained at 22 °C and 50 ± 10%, respectively. Protein rodent maintenance diet (Teklad HSD Inc., Madison, WI, USA) and drinking water were provided ad libitum. Mice were acclimated for 1 week before the start of the experiments. 

### 2.2. Gas Exposure

**Figure 1:**
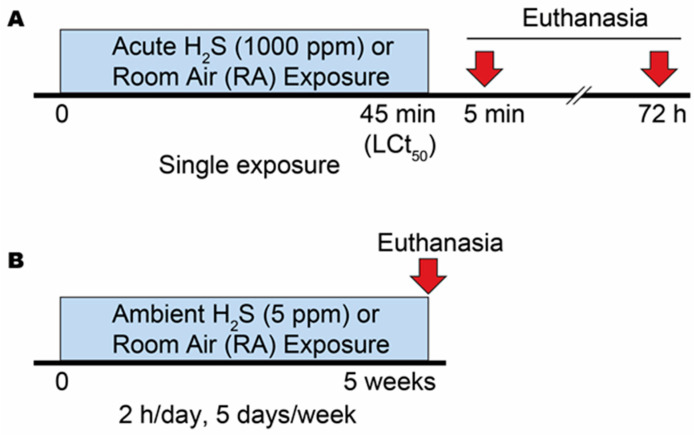
A summary of experimental exposure paradigms. (**A**) Exposure paradigm of single acute exposure to 1000 ppm H_2_S. This exposure model induces 50% mortality (LCt_50_). Control mice were exposed to room air. (**B**) Exposure paradigm of subchronic ambient exposure to 5 ppm H_2_S. In this model, mice were exposed to 5 ppm H_2_S for 2 h/day, 5 days/week for 5 weeks. Control mice were exposed to room air.

We conducted two separate experiments to mimic the two real-world scenarios of H_2_S exposure. In the first scenario, we conducted acute H_2_S exposure to mimic situations involving large scale industrial accidents or nefarious acts (Figure 1A). In the second scenario, we conducted subchronic exposure to low level ambient H_2_S to mimic occupational settings (Figure 1B). According to the OSHA guidelines, workers can be exposed to up to 10 ppm H_2_S for 8 h a day (TWA), 5 days a week without negatively impacting their health [22]. For this reason, we chose to use H_2_S at a concentration of 5 ppm. 

#### 2.2.1. Acute Exposure Experiment

For the acute study, we followed our previously published LCt_50_ model [3,5,6,7,8,9,12,13] (Figure 1A). Briefly, two cohorts of male mice (*n* = 10) were exposed to 1000 ppm of H_2_S for 45 min using a whole-body inhalation exposure chamber, as reported previously [3,5,6,7,8,9,12,13]. This level and length of exposure results in 50% mortality (lethal concentration time 50 [LCt_50_]) during exposure, and mice typically exhibit dyspnea, convulsions, loss of consciousness, and death in that order [9]. For the metabolomic analysis, we used mice that survived the LCt_50_ exposure. One cohort of surviving mice was sacrificed 5 min post exposure in order to determine the metabolic profile of mice immediately after exposure while the mice were still manifesting clinical effects of acute exposure. The other cohort was sacrificed 72 h post H_2_S exposure in order to determine metabolic changes at the time when neurodegenerative changes are typically initially observed in this model histologically [3,5,9]. For the controls, we used two cohorts of mice exposed once to normal room air (RA) for the same duration as H_2_S-exposed mice. As was the case for H_2_S-exposed mice, one cohort of control mice (*n* = 5) was euthanized at 5 min post RA exposure while the other was euthanized at 72 post RA exposure. All mice that received RA survived. Both H_2_S gas and RA were introduced to the exposure chamber from pressurized gas cylinders. The concentration of H_2_S in the exposure chamber was monitored in real time using a H_2_S sensor (RKI Eagle, RKI Instrument, Union City, CA, USA). Following exposure, mice were euthanized by decapitation and the brainstem (medulla and pons) was immediately further micro-dissected and the pontine reticular nucleus and the gigantocellular reticular nucleus isolated on ice. These tissue samples were immediately stored at −80 °C until metabolomic analysis.

#### 2.2.2. Ambient Exposure Experiment

In this experiment, male mice (*n* = 5) were exposed to 5 ppm H_2_S, 2 h/day, 5 days/week for 5 weeks, while the control group of mice (*n* = 4) were exposed to normal room air (RA). This dosage was chosen based on our prior pilot studies and also because 5 ppm is the midpoint concentration of the OSHA guideline of a time weighted average of 10 ppm of H_2_S in an 8 h work day, 5 days a week for the construction and shipyard industries [23]. Both H_2_S gas and RA were delivered to the exposure chambers from pressurized gas cylinders. None of the mice in this subchronic experiment died. The experimental design is shown in Figure 1B.

### 2.3. Metabolomic Analysis

The micro-dissected brainstem tissue samples were stored at −80 °C before submission to the West Coast Metabolomics Center, UC Davis, for untargeted metabolomics analysis. These metabolomic analyses consisted of three assays: (1) primary metabolism by GC-TOF MS, (2) biogenic amines (hydrophilic compounds) by HILIC-MS/MS, and (3) lipidomics by RPLC-MS/MS. Control samples from mice exposed to RA and samples from H_2_S-exposed mice were processed identically. Detailed procedures of metabolomic analysis is described in the supplementary data [24,25,26,27]. 

### 2.4. Data Analysis 

Raw data peak heights were normalized to the sum of internal standards for primary amine and lipidomics data analyses. Data of primary metabolites were normalized by a vector normalization method by calculating the sum of all peak heights of all identified metabolites. Data were further processed to identify outliers by using a boxplot with 1.5× interquartile range (IQR). Outliers were removed for the definitive analysis. The dataset of metabolomic analysis is publicly available in dryad, an open data publishing platform (https://doi.org/10.5061/dryad.tdz08kq5n) (accessed on 21 December 2023).

### 2.5. Enrichment Analysis

Quantitative metabolite set enrichment analysis (MSEA) was performed using MetaboAnalyst 5.0 (https://www.metaboanalyst.ca) (accessed on 21 December 2023). Metabolites that were significantly altered compared to the RA groups were further analyzed by quantitative MSEA. 

### 2.6. Heatmap

Metabolites that were significantly altered compared to the RA groups were summarized and visualized in a heatmap. Concentration changes in metabolites were visualized as a log 2-fold change. Visualization was processed using Python version 3.0 (https://www.python.org) accessed on 21 December 2023.

### 2.7. Statistical Analysis

Data are presented as mean and standard deviation of the mean. ANOVA with post hoc Tukey HSD test was performed for metabolites of acute H_2_S exposure using the statsmodels module in Python (https://www.python.org, version 3.0) accessed on 21 December 2023. Unpaired Student’s *t*-test was performed for the metabolites of the ambient H_2_S exposure study using Excel software of Microsoft 365. *p*-values ≤ 0.1 were considered statistically significant for this explorative analysis. Statistical analysis of the metabolite set enrichment data was performed in MetaboAnalyst 5.0 (https://www.metaboanalyst.ca) accessed on 21 December 2023.

## 3. Results

### 3.1. Exposure to Acute and Ambient Hydrogen Sulfide

Mice exposed acutely to H_2_S for 45 min manifested seizures, dyspnea, ataxia, and knockdown during exposure, as previously reported [3,5,6,7,9,12,13]. Mice that survived after acute H_2_S exposure and were euthanized at 5 min post H_2_S exposure exhibited weak, ataxic, and labored breathing. Mice euthanized at 72 h post H_2_S exposure were active and moved about normally. However, mice acutely exposed to H_2_S exhibited a reduction in body weight [3]. In contrast, mice from the 5 ppm exposure group did not exhibit any abnormal clinical signs or behavior, despite a reduction in body weight 30 days post ambient H_2_S exposure. 

### 3.2. Metabolomic Changes in the Brainstem following Acute and Ambient Hydrogen Sulfide

**Figure 2:**
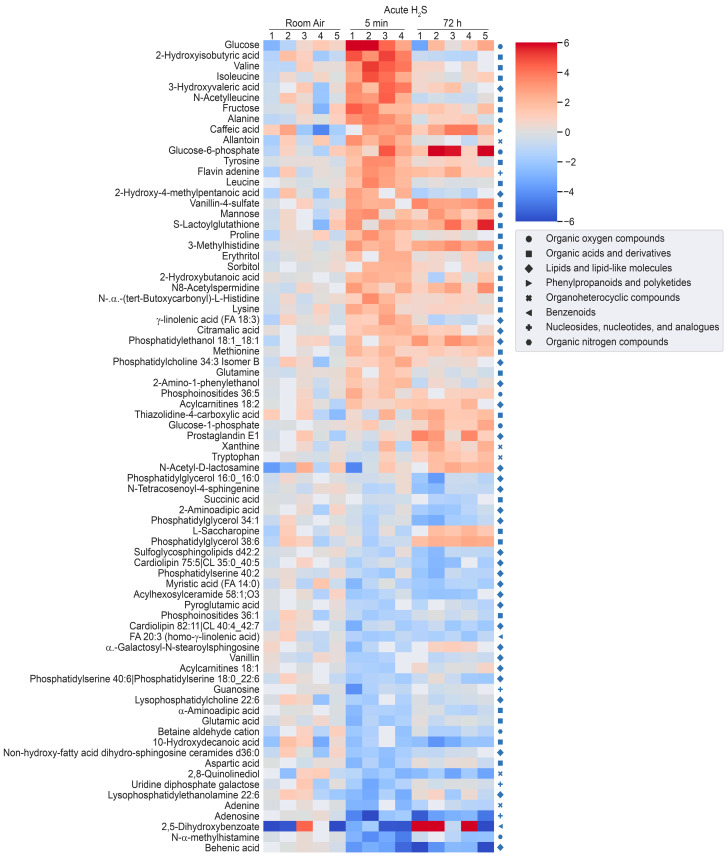
Heatmap analysis of significantly altered metabolites following a single acute H_2_S or RA exposure. Concentrations of significantly altered metabolites from primary metabolites, biogenic amines, and lipidomic metabolites are visualized in a heatmap by fold change (log_2_ scale). Each metabolite was compared to the average concentration of RA control group. Classification of individual metabolites is shown on the right side of the heatmap.

**Figure 3:**
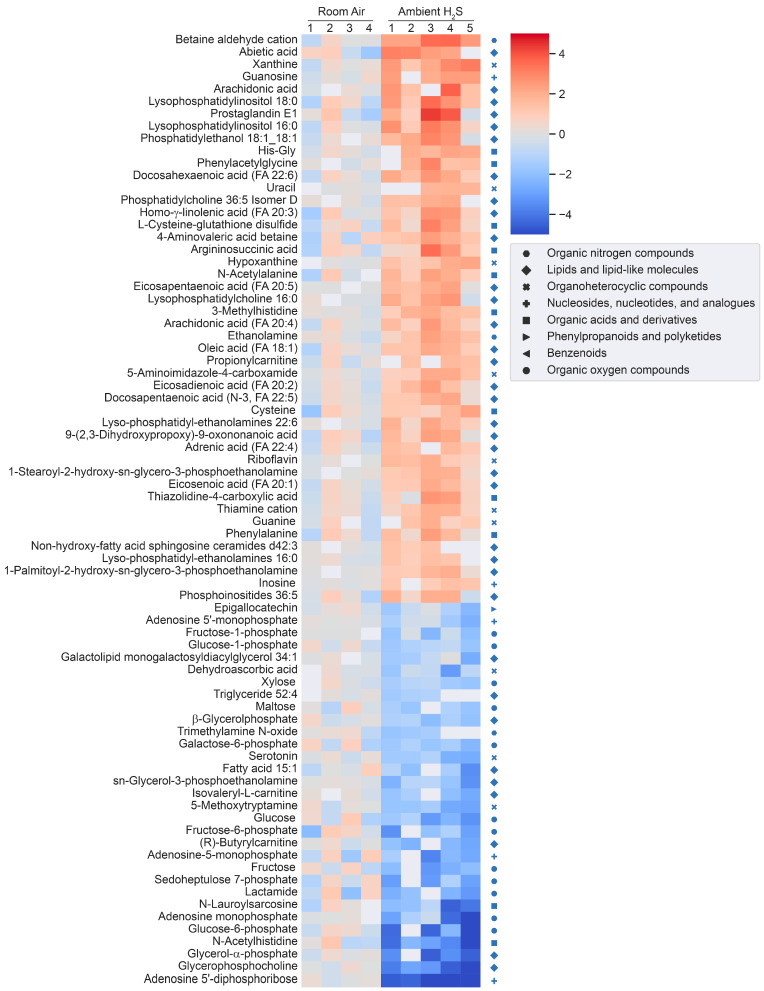
Heatmap analysis of significantly altered metabolites following subchronic ambient H_2_S or RA exposure. Concentrations of significantly altered primary organic metabolites, hydrophilic compounds including biogenic amines, and lipidomic metabolites are visualized in a heatmap by fold change (log_2_ scale). Each metabolite was compared to the average concentration of RA control group. Classification of individual metabolites is shown on the right side of the heatmap.

**Figure 4:**
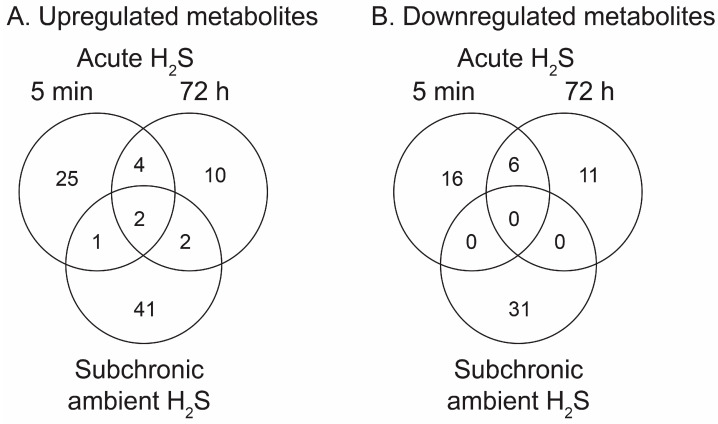
Venn diagrams summarizing numbers of significantly altered metabolites following acute and ambient H_2_S exposure. Immediate (5 min) and early (72 h) responses to acute H_2_S and response to ambient H_2_S exposure in brainstem are shown. (**A**) Increased metabolites compared to RA control group. (**B**) Decreased metabolites compared to RA control group.

H_2_S induced significant metabolomic changes in the brainstem. Metabolomic changes in brainstems from mice given a single high dose of acute exposure were different from those of mice repeatedly administered low levels of H_2_S over a month. The results of the changes from the metabolomic analyses included primary metabolites, biogenic amine metabolites, and lipidomic metabolites (summarized in heatmaps for acute and ambient H_2_S exposures; Figure 2 and Figure 3). The Venn diagram shows the number of significantly altered identified metabolites which were increased more than 50% or decreased 33% compared to the room air control group (Figure 4). The Venn diagram represents the number of significantly altered metabolites in common or which were unique at 5 min and at 72 h post acute H_2_S exposure and following subchronic ambient H_2_S exposures. The significantly altered metabolites are listed in Table 1 and Table 2 for 5 min and 72 h post acute H_2_S exposure and following subchronic ambient H_2_S exposure, respectively.

In the Venn diagram, immediate response reflects metabolic changes in brainstems of mice acutely exposed to H_2_S and euthanized 5 min post acute exposure, while early response reflects changes at 72 h post acute exposure. The results show that both acute and subchronic H_2_S exposure significantly altered brainstem metabolites. Several metabolites including a phosphatidylinositol (PI 36:5), prostaglandin E1, thiazolidine-4-carboxylic acid, 3-methyhistidine, and phosphatidylethanol 18:1_18:1 (PEtOH 18:1_18:1) were increased both at 5 min post acute H_2_S and subchronic ambient H_2_S, while no common metabolites were decreased by both acute and subchronic ambient H_2_S exposures relative to the RA group (Figure 4B). 3-Methylhistidine and PEtOH 18:1_18:1 were increased at all three euthanasia time points. Metabolites which increased in common due to acute H_2_S exposure both at 5 min and 72 h include caffeic acid, *N*^8^-acetylspermidine, vanillin-4-sulfate, flavin adenine, 3-methylhistidine, and PEtOH 18:1_18:1, while those which decreased due to acute H_2_S exposure were N-α-methylhistamine, 2,8-quinolinediol, vanillin, a glycerophospholipid (Cardiolipin 40:4_42:7), adenosine, and behenic acid. Metabolites presented in the heatmap analyses are further listed in Table 1 and Table 2 with mass-to-charge ratio and fold change (log_2_ scale).

### 3.3. Enrichment Analysis

**Figure 5:**
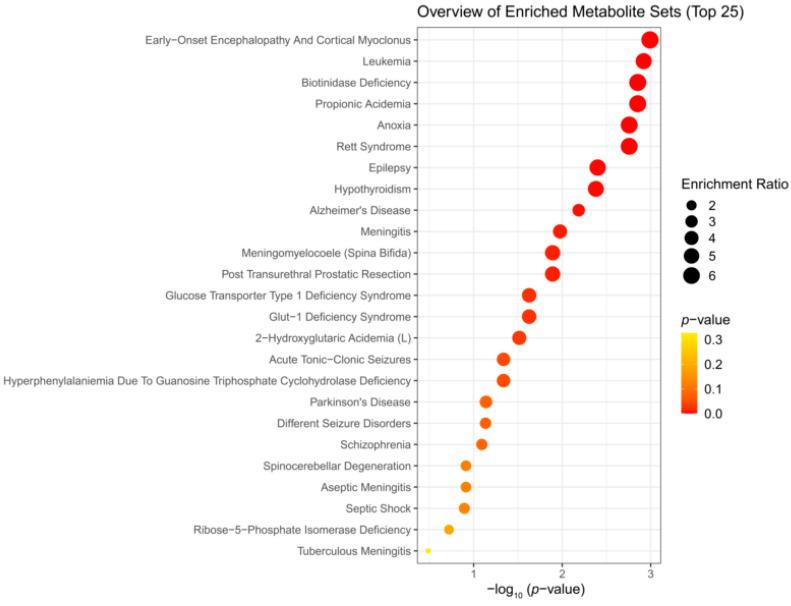
Disease-based enrichment analysis of significantly altered metabolites of immediate response to H_2_S exposure (5 min post single acute H_2_S exposure) for primary metabolites, biogenic amines, and lipidomic metabolites.

**Figure 6:**
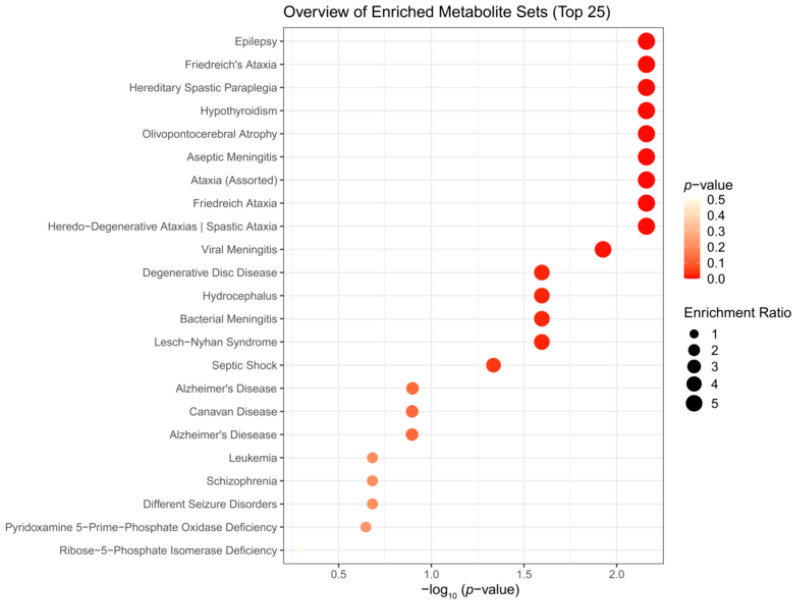
Disease-based enrichment analysis of significantly altered metabolites of early response 72 h post single acute H_2_S exposure for primary metabolites, biogenic amines, and lipidomic metabolites.

**Figure 7:**
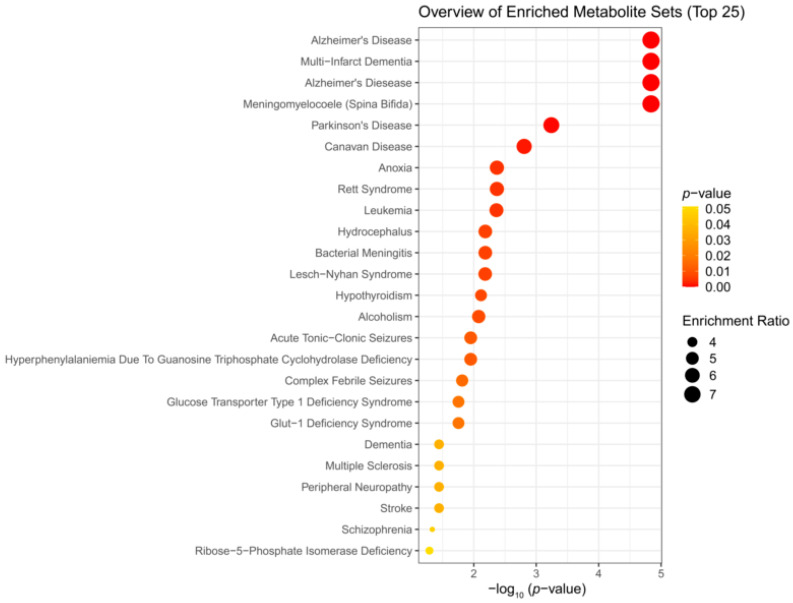
Disease-based enrichment analysis of significantly altered metabolites of subchronic response to repeated H_2_S exposure for 5 weeks for primary metabolites, biogenic amines, and lipidomic metabolites.

We performed enrichment analysis using Metaboanalyst 5.0 to investigate whether H_2_S-induced metabolic profiles cause any biological dysregulation and whether H_2_S-induced metabolic dysregulations share any similarity with any existing pathological signature databases in Metaboanalyst 5.0. While the database did not have brainstem as a tissue, we chose the human cerebral spinal fluid (CSF) dataset as this most closely matched our experimental design. The results showed several matches with human neuro-pathological conditions which are summarized in Figure 5, Figure 6 and Figure 7. Briefly, the metabolomic profiles following acute H_2_S poisoning matched with several neurological disorders including seizures, epilepsy, Alzheimer’s disease, and Glut-1 deficiency syndrome, among others (Figure 5 and Figure 6). The metabolomic profiles of subchronic ambient H_2_S exposure matched with several pathological conditions including Alzheimer’s disease, dementia, Parkinson’s disease, seizures, Glut-1 deficiency, and anoxia, among others (Figure 7).

### 3.4. H_2_S Dysregulated Brainstem Neurotransmitters

**Figure 8:**
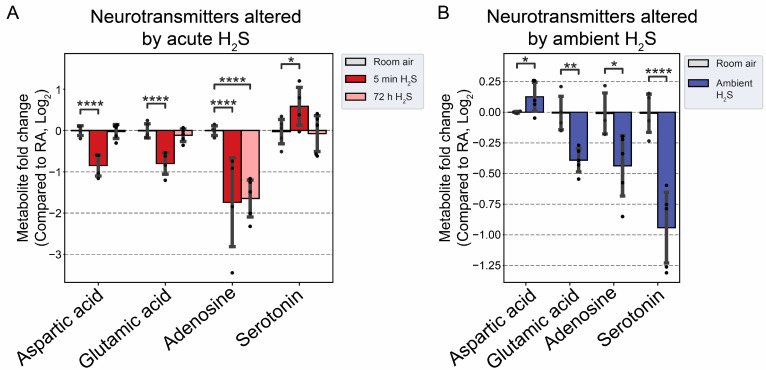
Neurotransmitter changes following H_2_S exposure. (**A**) Fold change in neurotransmitters following acute 1000 ppm H_2_S exposure. (**B**) Fold change in neurotransmitters following subchronic ambient H_2_S exposure. H_2_S groups were compared to RA control group. Values are presented as mean ± standard deviation. ANOVA with post hoc Tukey HSD test was performed for acute H_2_S exposure groups, while unpaired Student’s *t*-test was performed for subchronic ambient H_2_S exposure groups to test for statistical significance. Asterisks indicate significant differences compared to RA control group. *p* * < 0.1, ** < 0.05, and **** < 0.001.

**Figure 9:**
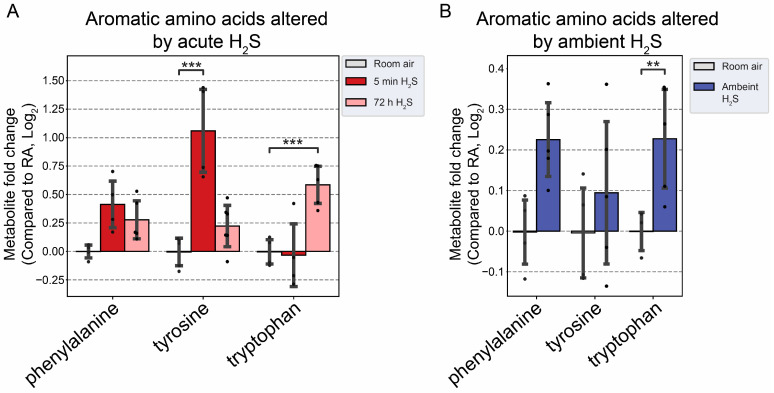
Altered aromatic amino acids following H_2_S exposure. (**A**) Fold change in aromatic amino acids following acute 1000 ppm H_2_S exposure. (**B**) Fold change in aromatic amino acids following subchronic ambient H_2_S exposure. H_2_S groups were compared to RA control group. Values are presented as mean ± standard deviation. ANOVA with post hoc Tukey HSD test was performed for acute H_2_S exposure groups, while unpaired Student’s *t*-test was performed for subchronic ambient H_2_S exposure groups to test for statistical significance. Asterisks indicate statistically significant differences compared to RA control group. *p* ** < 0.05 and *** < 0.01.

We analyzed neurotransmitters following both acute and subchronic ambient H_2_S exposures in the brainstem region. Interestingly, excitatory neurotransmitters, namely, aspartate and glutamate, were decreased, while serotonin, an inhibitory neurotransmitter, was increased at 5 min after single acute H_2_S exposure (Figure 8A). Adenosine, which acts both as a central excitatory and as an inhibitory neurotransmitter in the brain, was decreased following both acute and subchronic H_2_S exposures. Subchronic ambient H_2_S exposure decreased serotonin concentration in the brainstem (Figure 8B). Tyrosine, a precursor of catecholamines (excitatory), was also increased at 5 min post acute H_2_S exposure, while tryptophan, a precursor of serotonin, was increased at 72 h post acute H_2_S exposure. Tryptophan was also increased due to subchronic ambient H_2_S exposure (Figure 9B). 

### 3.5. H_2_S Dysregulated Energy Homeostasis

**Figure 10:**
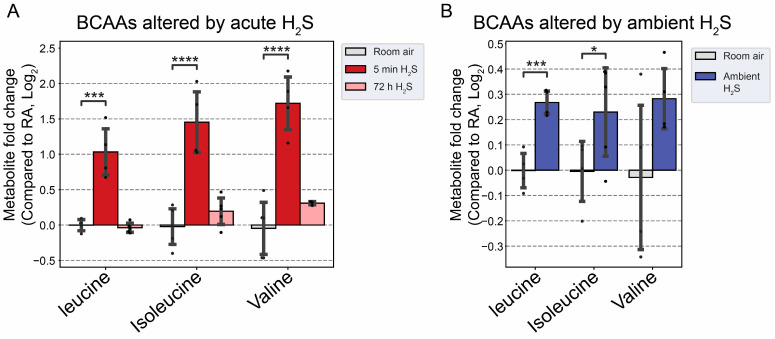
Fold change in branched-chain amino acids following H_2_S exposure. (**A**) Fold change in BCAAs following acute H_2_S exposure. (**B**) Fold change in BCAAs following subchronic ambient H_2_S exposure. H_2_S groups were compared to RA control group. Values are presented as mean ± standard deviation. ANOVA with post hoc Tukey HSD test was performed for acute H_2_S exposure groups, while unpaired Student’s *t*-test was performed for subchronic ambient H_2_S exposure groups for statistical significance. Asterisks indicate significant differences compared to RA control group. *p* * < 0.1, *** < 0.01, and **** < 0.001.

We analyzed to determine changes in lipid metabolism in the brainstem following acute and subchronic H_2_S exposures. Immediately following acute H_2_S exposure, BCAAs were significantly increased more than two-fold (Figure 10A). Interestingly, subchronic ambient H_2_S also significantly increased BCAAs, albeit less than acute exposure (Figure 10B). 

**Figure 11:**
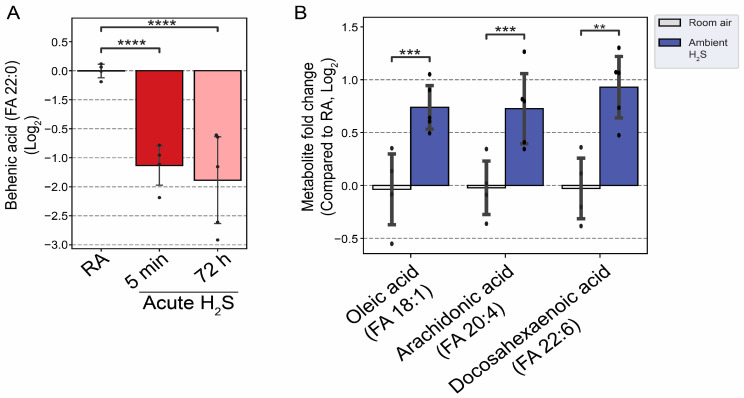
Altered fatty acids following H_2_S exposure. (**A**) Decreased behenic acid, a very long-chain fatty acid (VLCFA), following acute H_2_S exposure. (**B**) Major unsaturated fatty acids in brain were increased following subchronic ambient H_2_S exposure. H_2_S groups were compared to RA control group. Values are presented as mean ± standard deviation. ANOVA with post hoc Tukey HSD test was performed for acute H_2_S exposure groups, while unpaired Student’s *t*-test was performed for subchronic ambient H_2_S exposure groups for statistical significance. Asterisks indicate significant differences compared to RA control group. *p* ** < 0.05, *** < 0.01, and **** < 0.001.

Behenic acid concentration, a very long-chain fatty acid, was significantly decreased more than two-fold following acute H_2_S exposure (Figure 11A). Following subchronic ambient H_2_S exposure, major fatty acids in the brainstem including oleic acid, arachidonic acid (AA), and docosahexaenoic acid (DHA) were significantly increased compared to control (Figure 11B). 

**Figure 12:**
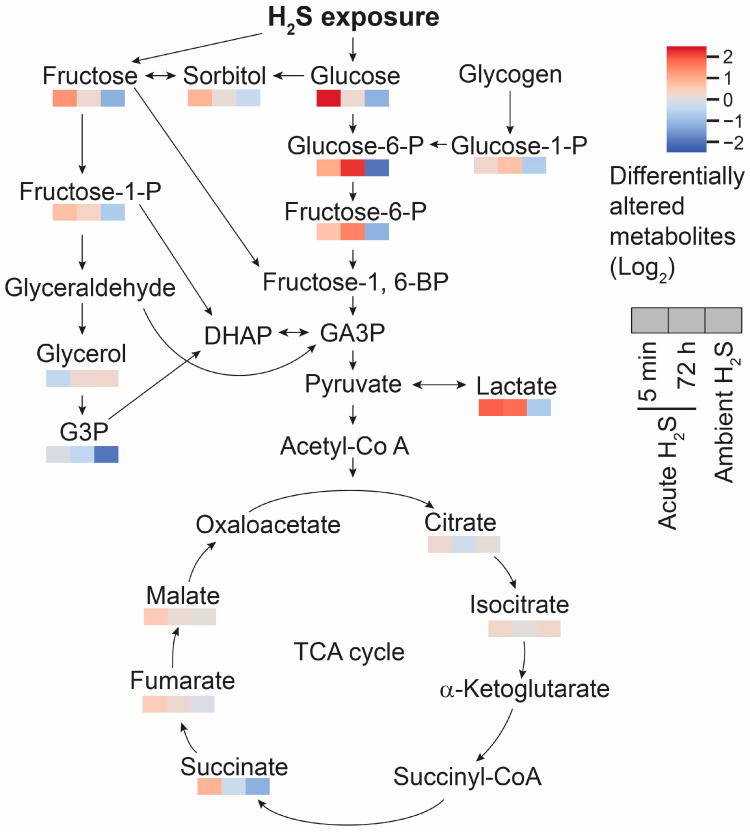
Schematic pathway of glycolysis and TCA cycle. Responses at 5 min and 72 post acute H_2_S exposure and also following subchronic ambient H_2_S are presented in a heatmap analysis. Fructose, sorbitol, and glucose were significantly increased by acute H_2_S exposure but decreased by subchronic ambient H_2_S exposures compared to RA control group. Changes in lactic acid are shown in a similar fashion. Succinate, fumarate, and malate concentrations were increased at 5 min post acute H_2_S exposure. Differentially altered metabolites are presented in fold changes (log_2_ scale). Abbreviations: DHAP: Dihydroxyacetone phosphate; Fructose-1-P: Fructose 1-phosphate; Fructose-1,6-BP: Fructose 1,6-bisphosphate; Fructose-6-P: Fructose 6-phosphate; G3P: Glycerol-3-phosphate; GA3P: Glyceraldehyde 3-phosphate; Glucose-1-P: Glucose 1-phosphate; Glucose-6-P: Glucose 6-phosphate; and TCA cycle: Ttricarboxylic acid cycle.

Cellular respiration was also dysregulated following both acute and subchronic ambient H_2_S exposures. Glucose concentration was significantly elevated at 5 min post acute H_2_S exposure, while it decreased following ambient H_2_S exposure (Figure 12). Elevated glucose concentration can affect multiple biological pathways. The polyol pathway converts glucose to sorbitol and then to fructose. Sorbitol and fructose concentrations were also significantly increased at 5 min post acute H_2_S exposure. Lactate is formed via glycolysis and was also increased following acute H_2_S exposure, but it was decreased following subchronic ambient H_2_S exposure. However, ribulose 5-phosphate and UDP-GlcNAc, intermediates of the pentose phosphate pathway (PPP) and hexosamine biosynthetic pathways (HBP), respectively, were not significantly altered. Succinate, fumarate, and malate which are intermediates of the Krebs cycle were also increased at 5 min post acute H_2_S exposure.

### 3.6. H_2_S Dysregulated Inosine Metabolism

**Figure 13:**
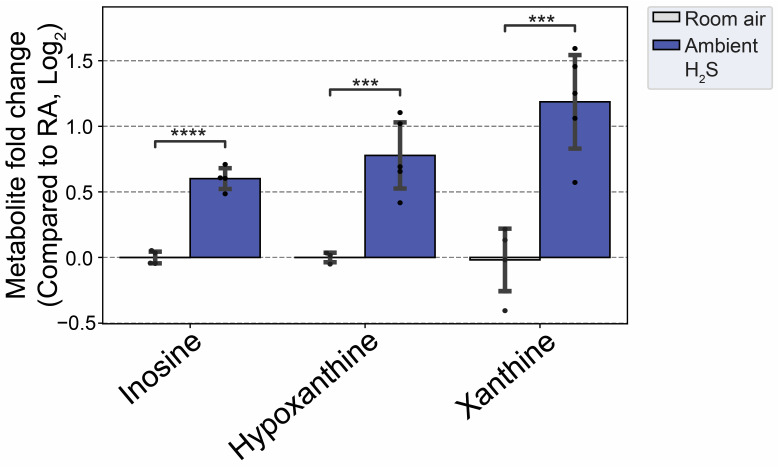
Altered inosine and metabolites following subchronic ambient H_2_S exposure. H_2_S groups were compared to RA control group. Values are presented as mean ± standard deviation. ANOVA with post hoc Tukey HSD test was performed for acute H_2_S exposure groups, while unpaired Student’s *t*-test was performed for subchronic ambient H_2_S exposure groups for statistical significance. Asterisks indicate significant differences compared to RA control group. *p* *** < 0.01 and **** < 0.001.

Inosine and its metabolites (hypoxanthine and xanthine) were increased by subchronic ambient H_2_S exposure (Figure 13), indicating an increased catabolism of purines under these conditions. Allantoin, an oxidation product of urate and usually considered a marker of oxidative stress, was the only metabolite increased at 5 min post acute H_2_S exposure. In contrast, inosine, hypoxanthine, and xanthine were all increased following subchronic ambient H_2_S exposure along with guanine and guanosine which are converted to xanthine (Figure 13).

## 4. Discussion

Hydrogen sulfide is widely reported to impact a broad spectrum of physiological functions. It is also reported to be a highly toxic compound with steep dose–response relationships. Acute high dose exposures are linked to acute death and to permanent neurobehavioral abnormalities and neurodegeneration [28,29,30,31], while the impacts of chronic low level H_2_S exposure in humans remain ambiguous. Acute H_2_S exposure impairs breathing and this is widely cited as the cause of H_2_S-induced acute death. The brainstem is an important brain region which regulates autonomous functions including breathing [32]. Despite the significance of this compound in health and disease, the lack of data on the impact of H_2_S on the brain metabolism is striking. This is the first comprehensive metabolomic study of acute high dose H_2_S exposure and subchronic low dose H_2_S exposure on the brain. For the subchronic low dose study, we used 5 ppm H_2_S to expose mice only for 2 h per day Monday through Friday for 5 weeks. As a reference, the OSHA TWA exposure guidelines for an 8 h workday, 5 days a week, is 10 ppm. The results showed that both acute and subchronic ambient H_2_S significantly altered many classes of metabolites including organic oxygen compounds, organic acids, phenylpropanoids, polyketides, organoheterocyclic compounds, benzenoids, nucleosides, nucleotides, organic nitrogen compounds, lipids, and lipid-like molecules in the brain. Importantly, metabolic changes induced by H_2_S exposure were different depending on whether it was a high dose single exposure or subchronic low dose exposure. Some metabolites, however, were increased both by acute and by subchronic exposures, suggesting that such compounds are sensitive indicators of H_2_S exposure. 

3-Methylhistidine, a derivative of histidine and methionine amino acids, and phosphatidylethanol 18:1_18:1 (PEtOH 18:1_18:1), a glycerophosphoethanol, were both significantly increased following both single H_2_S exposure and subchronic ambient H_2_S exposure. The functions of these two metabolites in the brainstem are not clear at the moment. Vanillin was decreased, while vanillin-4-sulfate was increased by acute H_2_S exposure. Vanillin has been linked to colorectal cancer. The roles of vanillin and vanillin-4-sulfate and the role of H_2_S exposure in altering vanillin and vanillin-4-sulfate remain to be determined. However, these metabolites may serve as potential biomarkers of H_2_S poisoning.

Several metabolites were altered at 5 min and 72 h following single acute H_2_S exposure. N^8^-acetyl spermidine was increased by 0.8-fold following acute H_2_S exposure. It is a polyamine derived from spermidine by deacetylation and is reported to play a role in regulating cell signaling and gene expression, among other functions [33]. It was previously reported that increases in N^8^-acetyl spermidine are caused by inhibiting N^8^-acetylspermidine deacetylase. This compound is linked to the differentiation of neurons and to elevated dopamine [33]. Interestingly, acute H_2_S exposure increases dopamine concentration in the brainstem [3]. N^8^-acetyl spermidine is also considered a biomarker for ischemic cardiomyopathy [34]. Interestingly, H_2_S is a cytochrome c oxidase inhibitor and causes chemical ischemia [35]. The exact role of N^8^-acetyl spermidine remains to be studied, but it may signal brain ischemia.

Flavin adenine, a derivative of riboflavin or vitamin B2, is also known as flavin adenine dinucleotide (FAD), and it plays a critical role as a cofactor of many enzymes. FAD was increased more than 1-fold at 5 min and 0.5-fold after 72 h following single acute exposure and is therefore a potential biomarker of acute H_2_S exposure in the brainstem. Since FAD plays a significant role as a cofactor in brain energy metabolism, it is possible that a sudden increase in FAD in the brainstem may indicate an imbalance in the redox cellular state (favoring FAD over FADH). FADH is not detectable by any of the mass spectrometers used in this study.

Adenosine and behenic acid were decreased 1-fold and 2-fold at 5 min and 72 h post acute H_2_S exposure, respectively. Adenosine is a nucleoside which plays many important roles in energy transfer, in signal transduction, as a neurotransmitter, and as a potent vasodilator [36]. H_2_S is well known to reduce ATP generation by inhibiting cytochrome c oxidase in the electron transport chain. Adenosine 5′-monophosphate was significantly decreased by about 1.5-fold following subchronic ambient H_2_S exposure. A decrease in adenosine concentration may limit several biological functions in which it is involved. Behenic acid, in contrast, is a very long-chain saturated fatty acid (VLCFA) and was decreased by more than 1.5-fold at 5 min and 72 h post acute H_2_S exposure, but not by subchronic H_2_S exposure. The reduction in behenic acid concentration may negatively impact energy production and homeostasis, among other effects. These results suggest that both acute and subchronic H_2_S exposure alter brainstem metabolism but in different ways. More research is needed to understand the implications of these results on brain function.

The brainstem has been reported to be particularly vulnerable to acute H_2_S poisoning. Following exogenous exposure, H_2_S was reportedly found in its highest concentrations in this brain region [20]. This brain region is also recognized as the center of regulation for key autonomic functions [37,38]. We have also previously shown that the brainstem is susceptible to H_2_S-induced neurodegeneration [3]. In addition, we showed that the inhibition of central respiratory drive (from the brainstem) is a key cause of death in our mouse model of acute H_2_S poisoning [3]. Clinical effects of acute H_2_S poisoning characterized by seizures and apnea, among others, were not seen in mice subchronically exposed to 5 ppm H_2_S for 1 month, indicating that the mechanisms involved were different. In this study, the only clinical effects noted following subchronic H_2_S exposure in mice was a loss in body weight. 

The results of the metabolite enrichment analysis summarized in Figure 5, Figure 6 and Figure 7 are very interesting. When the metabolomic profiles of acute and subchronic H_2_S exposure in mice are compared to existing databases of metabolomics of the cerebrospinal fluid from patients suffering from different neurological conditions, a pattern of similarities and differences emerges. At 5 min post exposure, the metabolomic enrichment pattern of acute H_2_S poisoning matches with many human neurological disorders including early-onset encephalopathy and cortical myoclonus, leukemia, propionic acidemia, anoxia, Rett syndrome, epilepsy, Alzheimer’s disease (AD), Parkinson’s disease (PD), Glut-1 deficiency syndrome, different seizure disorders, and schizophrenia, among others. In this mouse model, we have reported convulsions and neurodegeneration [3,7,8,9,12,13]. AD and PD are neurodegenerative diseases, while epilepsy matched with convulsions in our mouse model. We have also reported a significant increase in dopamine and serotonin concentrations in this model [3,9,12]. Dopamine is a modulatory neurotransmitter which is both excitatory and inhibitory depending on which receptors it binds to. Serotonin is predominantly inhibitory and has been linked to schizophrenia. The summary of neurotransmitter changes in Figure 8 suggests that acute and subchronic H_2_S exposure tilts neurotransmitters toward inhibitory and excitatory effects, respectively. It is probable that the loss of central respiratory drive is linked predominantly to the neurotransmitter status observed following acute H_2_S exposure in this study.

H_2_S exposure is widely reported to cause hypoxic neuronal injury, which is consistent with its mechanism of action of inhibiting *cytochrome c oxidase* activity [35]. Reduced *cytochrome c oxidase* activity is also reported in Alzheimer’s disease [39], and chronic neurodegenerative diseases are characterized by brain energy deficits [1,2]. The metabolomic profiles at 5 min post acute H_2_S exposure matched a metabolic profile of neurological disorders observed at 72 h min post acute exposure, including epilepsy, aseptic meningitis, Alzheimer’s disease, schizophrenia, and different seizure disorders. 

We were surprised to see that subchronic H_2_S exposure to 5 ppm, which is within the OSHA guidelines of 10 ppm for an 8 h workday, caused metabolic derangement in the brainstem. This is a significant finding which needs confirmation because of the health implications in the workplace. Strikingly, the metabolome of subchronic ambient H_2_S also matched with many human neurological disorders, including Alzheimer’s disease, Parkinson’s disease, and schizophrenia, which was similar to what we observed after single acute H_2_S exposure, with some exceptions. Notably, the metabolome of subchronic ambient H_2_S exposure additionally matched that of the CSF from patients with dementia, Canavan disease, alcoholism, and multiple sclerosis. Common themes of both acute and subchronic low dose H_2_S exposure revolve around metabolomic similarities between the metabolome of H_2_S exposure and that of seizure disorders and neurodegenerative conditions, especially Alzheimer’s disease, Parkinson’s disease, neuroinflammation, ataxia, and hypoxic-induced injury. This possibly signals that environmental H_2_S exposure shifts the brain metabolism, which may predispose or increase susceptibility to these debilitating disease conditions. Indeed, this mouse model with acute H_2_S exposure exhibited seizures, knockdown, and acute death [3,6,7,8,9,12,13]. These results signal that H_2_S-induced metabolomic changes play important roles in H_2_S-induced physiological symptoms including seizures and loss of consciousness. Notably, it is interesting that patients predisposed to seizures or neurofibromatosis are very sensitive to acute H_2_S poisoning [40]. Also, H_2_S aggravated seizure-like events in a rat seizure model [41]. Regarding the discovery of similarities in the metabolomes of acute and subchronic H_2_S exposure to those of the CSF of patients with Alzheimer’s disease, there is meagre literature on this topic. It is well known that the interaction of genes and the environment plays important roles in many neurological disorders including Alzheimer’s disease and Parkinson’s disease. Considering that H_2_S is an environmental pollutant of occupational concern and that H_2_S exposure may be closely linked to many neurological disorders with big health and economic burdens in society, more work should be focused on this area to investigate the potential role of ambient H_2_S exposure in AD and/or other neurological conditions summarized in Figure 5, Figure 6 and Figure 7.

The effects of acute H_2_S and subchronic ambient H_2_S exposures on neurotransmitters were investigated. Acute H_2_S exposure decreased aspartic acid, glutamic acid, and adenosine, while serotonin was increased. Serotonin was significantly decreased by subchronic ambient H_2_S exposure. Aspartic acid and glutamic acid are excitatory neurotransmitters, while serotonin is inhibitory [42,43]. Adenosine acts both as an excitatory and inhibitory neurotransmitter. We previously reported that dopamine and serotonin were significantly increased in the brainstem at 5 min post acute H_2_S [3], which is consistent with the findings in this study. Tryptophan is a precursor to serotonin, while phenylalanine and tyrosine are precursors to dopamine. In this study, tyrosine was significantly increased at 5 min post acute exposure in the brainstem, while tryptophan was increased by subchronic ambient H_2_S exposure. Other neurotransmitters including acetylcholine, histidine, serine, noradrenaline, γ-aminobutyric acid (GABA), glycine, and histamine were not significantly altered in this study. 

Glutamate and aspartate are the main excitatory neurotransmitters in the brain and are released in a Ca^2+^-dependent manner upon electrical stimulation. Serotonin is an inhibitory neurotransmitter and has an impact on a wide range of behavioral effects such as appetite, aggression, memory, fear, and depression. It is also involved in other central nervous system (CNS) functions such as respiration, body temperature, motor control, and bowel movement [44]. It is well known that acute exposure to high concentrations of H_2_S induces breathing suppression, a loss of consciousness, and acute death in humans. However, the exact mechanisms behind these effects of acute H_2_S-induced toxicity are not known. A decrease in glutamate and aspartate coupled with an increase in serotonin following acute H_2_S exposure in the brainstem may play important roles in H_2_S-induced inhibition of breathing and/or loss of consciousness, considering that the brainstem regulates essential vital functions including breathing activity and consciousness [32]. Interestingly, we recently reported that sodium sulfide, a H_2_S donor, suppressed neuronal activity in vitro by suppressing Ca^2+^ oscillation in primary cortical neurons [6]. An electrical stimulus releases glutamate from synaptic vesicles to the synaptic cleft. The released glutamate is taken up to glial cells by glutamate–aspartate transporters and converted to glutamine, which is then transported back to the neuron. Glutaminase in mitochondria converts glutamine to glutamate and aspartate. Acute H_2_S exposure induced increased glutamine at 5 min post acute exposure, indicating that glutamate–glutamine cycling may be dysregulated. A decrease in aspartate may influence the conversion of glutamine to glutamate, leading to a decrease in glutamate upon acute H_2_S exposure. Serotonin was also reported to inhibit glutamate release and the action of released glutamate [43]. In addition, an acute surge of serotonin induces focal seizures [45].

Interestingly, subchronic ambient H_2_S exposure significantly decreased serotonin concentration. The current OSHA guideline sets the recommended airborne exposure limit (REL) to 10 ppm for hydrogen sulfide in the workplace [22]. To the best of our knowledge, it has not been previously reported that subchronic ambient H_2_S exposure dysregulates serotonin. Therefore, this finding is novel. There are many serotonin receptors. It should be interesting to investigate the health impact of decreased serotonin in subchronic ambient H_2_S exposure.

Glutamate not only acts as a neurotransmitter but also as an anaplerotic component in tricarboxylic acid (TCA) cycle in energy balance. Glutamate is converted to α-ketoglutarate. In addition to the altered glutamate and glutamine, fumarate and malate were increased by 0.5-fold compared to the RA control mice at 5 min post acute H_2_S exposure. Intermediates of the TCA cycle including succinate, fumarate, and malate were increased at 5 min post acute H_2_S exposure, while succinate and fumarate were decreased following ambient H_2_S exposure. 

Glucose is a key compound in glycolysis. Glucose concentration was significantly increased at 5 min post acute H_2_S exposure compared to the RA control group but decreased following ambient H_2_S exposure. An increase in brain glucose during acute H_2_S exposure may suggest that it is spared following the inhibition of cytochrome c oxidase by H_2_S. Elevated glucose concentrations can affect multiple biological pathways including the polyol pathway, glycolysis, the PPP, and HBP. Intermediates of the polyol pathway and glycolysis were increased, while the PPP and HBP were not significantly altered at 5 min post acute H_2_S exposure, indicating acute H_2_S exposure significantly dysregulates energy balance. An accumulation of succinate may be caused by inverse activity of succinate dehydrogenase converting fumarate to succinate [46]. Lactate concentration remained elevated following acute H_2_S exposure, which is in line with previous findings that the treatment of sodium hydrosulfide induced elevation in lactate. It was shown that acute H_2_S exposure-induced inhibition of cytochrome c oxidase in the brain, including the brainstem, remained up to 72 h [3]. It is plausible that the inhibition of cytochrome c oxidase subsequently affected the ETC and TCA cycle following acute H_2_S exposure, which could not meet the energy demand of the CNS. An accumulation of succinate was shown to produce ROS and induce static epilepticus in a kainic acid rat model [46]. Although the exact effects of ambient H_2_S exposure on decreased glucose levels needs to be confirmed, ambient H_2_S exposure decreased the glucose level compared to the RA control group. 

Fatty acids have multiple important roles in the nervous system, from serving as structural components of membranes, energy sources, signaling molecules, cellular differentiation, and apoptosis to contributing to pathological conditions such as aging and neurodegenerative diseases [47]. The regulation of fatty acids is tightly controlled and in different ways in different brain regions. Behenic acid is a VLCFA. VLCFAs are preferentially metabolized in the peroxisome. The statistically significant reduction in behenic acid observed in this study may indicate altered peroxisome fatty acid beta oxidation. Subchronic ambient H_2_S strikingly increased metabolites of both the mono-unsaturated fatty acid (MUFA) and poly-unsaturated fatty acid (PUFA) synthesis pathways in the brainstem, including oleic acid, arachidonic acid (AA), and docosahexaenoic acid (DHA) in the brainstem compared to the RA control group. Arachidonic acid, which was also increased, is pro-inflammatory and is also linked to neurodegenerative diseases such as AD [48]. A deficiency in DHA has been associated with neurodegenerative disorders [49]. We have also previously reported that acute H_2_S poisoning causes neuroinflammation and neurodegeneration [3,9,12,13]. 

Subchronic ambient H_2_S also dysregulated inosine metabolism and increased inosine, hypoxanthine, and xanthine. Inosine is metabolized to hypoxanthine, xanthine, and finally to uric acid [50]. Inosine was shown to play important roles in neuroprotection, among other roles, presumably via anti-inflammatory and antioxidant properties [51]. AMP was shown to be metabolized to IMP, hypoxanthine, xanthine, and uric acid to enhance ATP production during acute energy consumption [50]. The administration of inosine was shown to induce hypoglycemia [52]. Indeed, subchronic ambient H_2_S decreased glucose concentration more than 1-fold compared to the RA control group. Hypoxanthine and its metabolites were shown to be increased in post mortem tissues over a time span of several hours [53]. In this study, brainstem regions were immediately dissected and flash-frozen. Moreover, the changes in hypoxanthine and its metabolites were compared to those of the RA control group which were handled identically to brain tissues from H_2_S exposed mice. This finding shows that subchronic ambient H_2_S dysregulates energy homeostasis. More work is needed on this topic to determine whether subchronic ambient H_2_S exposure causes a brain energy deficit. Brain energy deficit has been cited a contributing factor in neurodegeneration [1,2]. Potentially, this suggests that ambient H_2_S exposure may be an environmental factor predisposing to neurodegeneration. 

This study has many potential applications. Understanding the basic mechanisms of H_2_S-induced neurotoxicity will lead to development of therapeutic drugs for the treatment of victims of acute and subchronic H_2_S poisoning. Hydrogen sulfide is considered a chemical weapon [54,55], and currently, there are no FDA-approved drugs for treating victims of acute H_2_S poisoning. Also, environmental pollutants, including those that affect mitochondria, play a role in inducing neurodegeneration. The results of this study will likely contribute to our knowledge on the role that H_2_S plays in neurodegeneration and other neurological diseases shown in Figure 5, Figure 6 and Figure 7. In addition, this work may guide the development of novel diagnostic and forensic biomarkers of acute and subchronic H_2_S poisoning. 

This study, though yielding interesting results, had some limitations. For example, this study only consisted of male, adult mice. Future studies should include female mice as well so as to determine whether there may be sex differences in brain metabolism following H_2_S exposure. As has been reported [9], male mice are more sensitive to acute H_2_S poisoning than females. Therefore, including female mice in this study may shed light on the toxic mechanisms of H_2_S. Another limitation of this study was that the acute study only lasted up to 72 h post H_2_S exposure. In previous studies, we have shown neurodegeneration to be fully manifested on day 7 in the brainstem and other brain regions [3,5,7,8,9]. Also, in human victims of single acute H_2_S poisoning accidents, a plethora of long-term debilitating neurological sequalae including neuropsychiatric disturbance, sleep disorders, headaches, memory and cognition deficits, and persistent vegetative states are reported [25,26,27,28,56]. Therefore, future studies should investigate metabolomic changes in the brainstem several months later to determine the metabolomic profiles of delayed neurotoxic effects. Finally, concentrations of tissue metabolites, especially brain tissue, can be influenced by and vary in sample collection and sample preparation procedures [57,58]. It has been shown that concentrations of brain tissue metabolites will decrease or increase if the post mortem autolysis is not prevented by procedures like microwave fixation before brain dissection [57,58]. In this study, control and H_2_S exposed mice were decapitated and the brain was rapidly removed and micro-dissected on ice over a 2–3 min time period before they were immediately stored at −80 °C until analysis. All experiments had suitable control samples and all samples were handled similarly, and comparisons were made between the RA control and H_2_S exposed mice. Thus, whereas the absolute concentrations of metabolites would likely be different if samples were microwave fixed before brain microdissection, the outcome of the study should be the same. However, future metabolomic studies will be performed on microwave-fixed mice to completely stop enzymatic activity during tissue collection and sample preparation. 

## 5. Conclusions

In summary, the results of this study identified 3-methylhistidine and PEtOH18:1_18:1 as potential biomarkers of H_2_S exposure, because they were significantly increased in the brainstems of mice exposed to H_2_S both acutely and subchronically. Twelve metabolites were altered by acute H_2_S exposure including N-acetylspermidine, adenosine, behenic acid, flavin adenine, vanillin, vanillin-4-sulfate, caffeic acid, and behenic acid, which were identified as potential biomarkers of acute H_2_S exposure. Acute H_2_S decreased glutamate and aspartate, while serotonin was increased, suggesting that overall acute H_2_S shifts neurotransmitters predominantly toward inhibitory status. Serotonin was decreased by subchronic ambient H_2_S exposure and so was adenosine, suggesting that chronic H_2_S exposure may predispose neurological excitation. Glucose and fructose were increased in acute H_2_S poisoning, while glucose was significantly decreased in subchronic ambient H_2_S. MUFAs and PUFAs were increased in subchronic ambient H_2_S exposure. Overall, the results of this study provide evidence that both acute and subchronic H_2_S exposure alters neurotransmitters and energy metabolism in the brainstem. This has opened novel mechanisms underlying H_2_S-induced neurotoxicity. Surprisingly, and more importantly, this study shows that current OSHA guidelines (10 ppm for 40 h/work week) may not be adequately protecting worker health, as a subchronic exposure of mice to 5 ppm H_2_S for only a few hours per day significantly altered neurotransmitters and energy metabolism.

## Figures and Tables

**Table 1:** Significantly altered metabolites at 5 min post acute H_2_S exposure compared to RA control group. Common metabolite name, mass-to-charge ratio, fold change (log_2_ scale), and statistical significance are listed. ANOVA with post hoc Tukey HSD test was performed for statistical significance. Asterisks indicate significant differences compared to RA group. *p* * < 0.1, ** < 0.05, *** < 0.01, and **** < 0.001.

Metabolites	*m*/*z*	5 min		72 h	
		Fold Change	Significance	Fold Change	Significance
10-hydroxydecanoic acid	187.135	1.77	****	0.31	
2,5-dihydroxybenzoate	329.025	1.52	****	0.21	
2,8-quinolinediol	162.057	1.46	***	0.32	
2-amino-1-phenylethanol	120.08	1.41	****	−0.2	
2-aminoadipic acid	162.076	1.36	***	0.48	
2-hydroxy-4-methylpentanoic acid	131.071	1.35	****	0.23	
2-hydroxybutanoic acid	131	1.22	*	1.18	**
2-hydroxyisobutyric acid	103.041	1.15	***	0.02	
3-hydroxyvaleric acid	117.056	1.15		2.88	*
3-methylhistidine	170.091	1.11	***	0.25	
Acylcarnitines 18:1	426.358	0.9	****	0.31	***
Acylcarnitines 18:2	424.344	0.87	***	0.19	
Acylhexosylceramide 58:1; O3	1048.91	1.07	***	−0.04	
Adenine	136.062	1.1		1.33	*
Adenosine	236	1.07	***	0.68	**
Alanine	116	1.01	***	−0.44	
Allantoin	157.038	1	***	1.29	****
α-galactosyl-N-stearoylsphingosine	728.598	1.79	****	−0.43	
α-aminoadipic acid	260	0.97	*	0.81	
Aspartic acid	232	0.96		1.49	**
Behenic acid	117	0.93	***	0.08	
Betaine aldehyde cation	120.1019_ 102.0893	0.93	***	1.19	****
Caffeic acid	181.057	0.91	****	0.13	
Cardiolipin 75:5|Cardiolipin 35:0_40:5	1496.04	0.79	***	0.29	
Cardiolipin 82:11|Cardiolipin 40:4_42:7	1582.09	0.78	**	−0.24	
Citramalic acid	247	0.83	**	0.26	
Erythritol	217	0.78	*	0.76	**
Flavin adenine	246	0.66	**	0.2	
Fructose	307	0.63	****	−0.06	
γ-Linolenic acid (FA 18:3)	277.216	0.69	*	1.06	***
Glucose	319	0.62	***	−0.02	
Glucose-1-phosphate	217	0.59	*	0.45	
Glucose-6-phosphate	387	0.47	*	0.65	**
Glutamic acid	246	0.27		0.8	*
Glutamine	156	0.26		0.7	**
Guanosine	324	0.12		1.07	**
Homo-gamma-linolenic acid (FA 20:3)	305.249	0.68	***	0.48	**
Isoleucine	158	0.09		0.71	*
Leucine	158	−0.06		0.87	*
L-Saccharopine	277.139	−0.23	***	−0.58	****
Lysine	317	−0.24		−0.62	**
Lysophosphatidylcholine 22:6	568.339	−0.07		−0.61	**
Lysophosphatidylethanolamine 22:6	526.288	−0.18		−0.58	**
Mannose	205	−0.24		−0.8	***
Methionine	150.056	−0.26		0.71	**
Myristic acid (FA 14:0)	227.202	0.75	***	0.47	**
N-(Octadecanoyl)-sphinganine (Cer-NDS d36:0)	566.547	0.86	*	0.88	*
N-α-(Tert-Butoxycarbonyl)-L-histidine	110.069	−0.27		0.93	****
N^8^-Acetylspermidine	188.175	−0.28		−0.6	**
N-Acetyl-D-lactosamine	406.13	−0.35		−0.9	***
N-Acetylleucine	172.096	−0.4		−0.66	**
N-α-Methylhistamine	126.101	−0.49		−0.68	*
N-tetracosenoyl-4-sphingenine	648.6272_ 630.6146	−0.5	*	−0.89	**
Phosphatidylcholine 34:3 Isomer B	756.5538_ 778.5289	−0.58	***	−0.39	***
Phosphatidylglycerol 16:0_16:0	721.502	−0.6	**	−0.6	**
Phosphatidylserine 40:2	844.603	−0.69	*	−0.19	
Phosphatidylserine 40:6|PS 18:0_22:6	836.547	−0.74	***	−0.29	*
Phosphoinositides 34:1	835.536	−0.61	**	−0.59	**
Phosphoinositides 36:1	863.56	−0.65	**	0.38	*
Phosphoinositides 36:5	855.5	−0.66	***	−0.3	
Phosphoinositides 38:6	881.512	−0.66	***	0.07	
Phostatidylethanol 18:1_18:1	727.527	−0.6	*	−0.19	
Proline	142	−0.66	*	−0.37	
Prostaglandin E1	353.232	−0.67	**	−0.16	
Pyroglutamic acid	130.05	−0.78	****	−0.11	
S-Lactoylglutathione	380.111	−0.82		−1.13	*
Sorbitol	217	−0.83	**	−0.3	
Succinic acid	117.019	−0.83	****	−0.02	
Sulfoglycosphingolipids d42:2	890.639	−0.8	*	−0.41	
Thiazolidine-4-carboxylic acid	134.025	−0.84	*	−1.05	**
Tryptophan	202	−0.87	**	−0.08	
Tyrosine	218	−1.06	*	−0.05	
Uridine diphosphate galactose	565.044	−1.18	****	−0.49	****
Valine	144	−1.42	****	−1.58	****
Vanillin	151.04	−1.53		4.94	*
Vanillin-4-sulfate	230.996	−1.54	****	−0.64	***
Xanthine	153.037	−1.6	****	−1.71	****

**Table 2:** Significantly altered metabolites following subchronic ambient H_2_S exposure compared to RA control group. Common metabolite name, mass-to-charge ratio, fold change (log_2_ scale), and statistical significance are listed. Unpaired Student’s *t*-test was performed for statistical significance. Asterisks indicate significant differences compared to RA group. *p* * < 0.1, ** < 0.05, *** < 0.01, and **** < 0.001.

Metabolites	*m*/*z*	Fold Change	Significance
(R)-Butyrylcarnitine	232.1547	−1.18	***
1-Palmitoyl-2-hydroxy-sn-glycero-3-phosphoethanolamine	454.2927	0.61	**
1-Stearoyl-2-hydroxy-sn-glycero-3-phosphoethanolamine	482.3181	0.68	**
3-Methylhistidine	170.0913	0.77	***
4-Aminovaleric acid betaine	160.133	0.8	**
5-Aminoimidazole-4-carboxamide	110.0322	0.72	**
5-Methoxytryptamine	174	−1.03	***
9-(2,3-Dihydroxypropoxy)-9-oxononanoic acid	261.1345	0.7	**
Abietic acid	301.216	1.34	***
Adenosine 5′-diphosphoribose	560.0761_582.0571	−3.46	****
Adenosine 5′-monophosphate	370.0487	−0.61	**
Adenosine monophosphate	695.1267	−1.48	**
Adenosine-5-monophosphate	315	−1.2	*
Adrenic acid (FA 22:4)	331.2637	0.69	***
Arachidonic acid	303.2328	1.13	*
Arachidonic acid (FA 20:4)	303.2333	0.77	**
Argininosuccinic acid	291.127	0.8	*
β-Glycerolphosphate	243	−0.76	***
Betaine aldehyde cation	120.1019_102.0893	1.4	***
Cysteine	220	0.71	**
Dehydroascorbic acid	173	−0.65	**
Docosahexaenoic acid (FA 22:6)	327.2328	0.96	***
Docosapentaenoic acid (n-3, FA 22:5)	329.2473	0.71	**
Eicosadienoic acid (FA 20:2)	307.2637	0.72	**
Eicosapentaenoic acid (FA 20:5)	301.2167	0.79	**
Eicosenoic acid (FA 20:1)	309.2798	0.68	**
Epigallocatechin	307.0835	−0.59	**
Ethanolamine	62.0596	0.76	**
Fatty acid 15:1	239.2009	−0.92	**
Fructose	307	−1.21	**
Fructose-1-phosphate	387	−0.62	**
Fructose-6-phosphate	315	−1.13	*
Galactolipid monogalactosyldiacylglycerol 34:1	774.605	−0.64	**
Galactose-6-phosphate	387	−0.82	*
Glucose	319	−1.11	**
Glucose-1-phosphate	217	−0.63	**
Glucose-6-phosphate	387	−1.9	***
Glycerol-alpha-phosphate	357	−1.97	****
Glycerophosphocholine	515.2116_772.3096_ 280.0915_258.1106_ 296.0648	−2.04	****
Guanine	152.0564	0.64	*
Guanosine	324	1.16	****
His-Gly	213.0949	1.01	****
Homo-γ-linolenic acid (FA 20:3)	305.2487	0.86	**
Hypoxanthine	265	0.8	***
Inosine	230	0.6	****
Isovaleryl-L-carnitine	246.17	−0.98	***
Lactamide	90.0544	−1.25	*
L-Cysteine-glutathione disulfide	427.0924	0.81	*
Lysophosphatidylcholine 16:0	540.3289	0.78	**
Lyso-phosphatidyl-ethanolamines 16:0	452.2776	0.62	****
Lyso-phosphatidyl-ethanolamines 22:6	526.2883	0.7	**
Lysophosphatidylinositol 16:0	571.2842	1.03	**
Lysophosphatidylinositol 18:0	599.3185	1.11	**
Maltose	361	−0.76	*
N-Acetylalanine	130.0515	0.79	*
N-Acetylhistidine	198.0888	−1.96	**
N-Lauroylsarcosine	272.2221	−1.35	*
Non-hydroxy-fatty acid sphingosine ceramides d42:3	704.6138	0.62	***
Oleic acid (FA 18:1)	281.2491	0.75	***
Phenylacetylglycine	192.0664	0.97	**
Phenylalanine	164.0721	0.62	*
Phosphatidylcholine 36:5 Isomer D	780.555	0.88	***
Phosphoinositides 36:5	855.5001	0.6	*
Phosphatidylethanol 18:1_18:1	727.5266	1.01	**
Propionylcarnitine	218.1366	0.75	***
Prostaglandin E1	353.2316	1.07	*
Riboflavin	377.1473	0.69	***
Sedoheptulose 7-phosphate	387	−1.24	**
Serotonin	174	−0.91	****
sn-glycerol-3-phosphoethanolamine	238.0433	−0.96	***
Thiamine cation	265.1075	0.65	**
Thiazolidine-4-carboxylic acid	134.0252	0.66	*
Triglyceride 52:4	872.7704	−0.67	***
Trimethylamine N-oxide	76.0748	−0.8	**
Uracil	241	0.89	****
Xanthine	353	1.23	***
Xylose	103	−0.66	*

## Data Availability

The data presented in this study are openly available in dryad at [https://doi.org/10.5061/dryad.tdz08kq5n] accessed on 21 December 2023.

## Supplementary Materials

The following supporting information can be downloaded at: https://www.mdpi.com/article/10.3390/metabo14010053/s1, Information S1: Primary metabolites by GC-TOF MS, Information S2: HILIC-MS/MS analysis for hydrophilic compounds including biogenic amines, Information S3: RPLC-MS/MS analyses for untargeted lipidomics metabolites.
