# Supplementary material for: Metabolomic Signatures of Brainstem in Mice following Acute and Subchronic Hydrogen Sulfide Exposure
Source: Metabolites. 2024 Jan 14;14(1):53. doi: 10.3390/metabo14010053 (PMC10819975; doi:10.3390/metabo14010053)
Supplement: Supplementary file 1 [file metabolites-14-00053-s001.zip › metabolites-2810703-supplementary.pdf]

Supplementary methods

S1. Primary metabolites by GC-TOF MS.

Tissues were prepared, samples analyzed, and data were acquired as previously reported [24] using derivatization with methoximation and trimethylsilylation. A Rtx-5Sil MS (30 m length x 0.25 mm internal diameter with 0.25 mm film, Restek corporation) was used with helium as carrier gas at 1mL/min flow rate. 0.5 µL of samples were injected in 25 s splitless mode at 250°C. Oven temperature was increased from 50°C (30s hold) at 20°C/min to 330°C with a 10 min hold time. A Leco Pegasus IV mass spectrometer was used with unit mass resolution at 17 spectra/s from 80-500 Da at -70eV ionization energy and 1800 V detector voltage with a 230 °C transfer line and a 250 °C ion source. ChromaTOF vs. 4.0 was used for data preprocessing without smoothing, 3 s peak width, baseline subtraction above the noise level, and automatic mass spectral deconvolution and peak detection at signal/noise levels of 5:1 throughout the chromatogram. Peak sorting and compound identification was performed in the BinBase database environment [24]. Quantification was reported as peak height of extraction ion chromatograms. Missing peaks were automatically replaced from ion traces of raw data, as detailed before [24]. Metabolites were reported by peak heights with common identifiers including common metabolite name, InChl key, PubChem ID, and KEGG ID.

S2. HILIC-MS/MS analysis for hydrophilic compounds including biogenic amines.

Samples were extracted in a biphasic solution [25]. The polar phase was used for hydrophilic interaction liquid chromatography (HILIC) used an Agilent 1290 UHPLC/Sciex Triple TOF 6600 mass spectrometer. HILIC was performed with a Waters BEH amide column (1.7 µm, 2.1 mm x 50 mm). 5 µL samples were injected into a mobile phase A (ultrapure water with 10 mM ammonium formate + 0.125% formic acid, pH 3), mobile phase B (95:5 v/v acetonitrile:ultrapure water with 10 mM ammonium formate + 0.125% formic acid, pH 3), at 45 °C for column temperature, at 0.5 mL/min with a gradient profile (100 % of mobile phase B at 0 min, 100 % of mobile phase B at 0.5 min, 70 % of mobile phase B at 1.95 min, 30 % of mobile phase B at 2.55 min, 30 % of mobile phase B at 3.15 min, 100% of mobile phase B at 3.8min). Chromatograms first underwent a quality control check with internal standards for consistency of peak height and retention time, followed by processing raw data files using MS-DIAL software [26] which identifies and aligns peaks and annotates peaks using both an in-house mzRT library and MS/MS spectral matching with NIST/MoNA libraries. All MS/MS annotations were manually curated, and only metabolites with high-quality compound identification were reported. The metabolites were reported by peak heights with common identifiers including common metabolite name and InChl key. Data were presented as peak heights for the quantification ion at the specific retention time.

S3. RPLC-MS/MS analyses for untargeted lipidomics metabolites.

Samples were extracted in a biphasic solution [25]. The lipophilic phase samples were used for separation on a BEH C18 column (50 mm x 2.1 mm internal diameter; 1.7 µm particles, Waters) at 0.8 mL/min flow rate with 65 °C for column temperature. For positive mode electron spray ionization (ESI), mobile phase A (60:40 v/v acetonitrile:water + 10 mM ammonium formate + 0.1

% formic acid) and mobile phase B (90:10 v/v isopropanol:acetonitrile + 10 mM ammonium formate + 0.1 % formic acid) was used as reported previously [27]. Negative mode ESI was performed with mobile phase A (60:40 v/v acetonitrile:water + 10 mM ammonium acetate) and mobile phase B (90:10 v/v isopropanol:acetonitrile + 10 mM ammonium acetate). Sample of 1.67 uL and 5 uL were injected at 4°C for positive electrospray ionization (ESI) and negative ESI, respectively. ESI capillary voltage was applied at +3.5 kV and -3.5 kV for positive and negative ESI, respectively. Collision energy was applied for 25 eV for both positive and negative ESI. Precursor/product isolation width was 4 Da. Scan range for positive mode was m/z 120-1200 Da while scan range for negative mode was m/z 60-1200 Da. Spectra were acquitted at 2 spectra/s. Positive mode used 10,000 mass resolution on an Agilent 6530 QTOF MS (Agilent), whereas negative mode used 20,000 mass resolution on an Agilent 6550 QTOF MS (Agilent). Data was processed using MS-DIAL as given above.

Reference

24. Fiehn, O. Metabolomics by Gas Chromatography-Mass Spectrometry: Combined Targeted and Untargeted Profiling. *Curr Protoc Mol Biol* **2016**, *114*, 30 34 31-30 34 32. DOI: 10.1002/0471142727.mb3004s114 From NLM Medline.

25. Matyash, V.; Liebisch, G.; Kurzchalia, T. V.; Shevchenko, A.; Schwudke, D. Lipid extraction by methyl-tert-butyl ether for high-throughput lipidomics. *Journal of lipid research* **2008**, *49* (5), 1137-1146. DOI: 10.1194/jlr.D700041-JLR200 From NLM Medline.

26. Tsugawa, H.; Cajka, T.; Kind, T.; Ma, Y.; Higgins, B.; Ikeda, K.; Kanazawa, M.; VanderGheynst, J.; Fiehn, O.; Arita, M. MS-DIAL: data-independent MS/MS deconvolution for comprehensive metabolome analysis. *Nature methods* **2015**, *12* (6), 523-526. DOI: 10.1038/nmeth.3393 From NLM Medline.

27. Cajka, T.; Smilowitz, J. T.; Fiehn, O. Validating Quantitative Untargeted Lipidomics Across Nine Liquid Chromatography-High-Resolution Mass Spectrometry Platforms. *Analytical chemistry* **2017**, *89* (22), 12360-12368. DOI: 10.1021/acs.analchem.7b03404 From NLM Medline.
